# Resonance Raman Studies on Heme Ligand Stretching Modes in Methionine80-Depleted Cytochrome *c*: Fe–His, Fe–O_2_, and O–O Stretching Modes

**DOI:** 10.1021/acs.jpcb.3c00514

**Published:** 2023-03-15

**Authors:** Mohan Zhang, Hulin Tai, Sachiko Yanagisawa, Masaru Yamanaka, Takashi Ogura, Shun Hirota

**Affiliations:** † Division of Materials Science, Graduate School of Science and Technology, Nara Institute of Science and Technology (NAIST), 8916-5, Takayama, Ikoma, Nara 630-0192, Japan; ‡ Graduate School of Life Science, University of Hyogo, Kamigori-cho, Ako-gun, Hyogo 678-1297, Japan

## Abstract

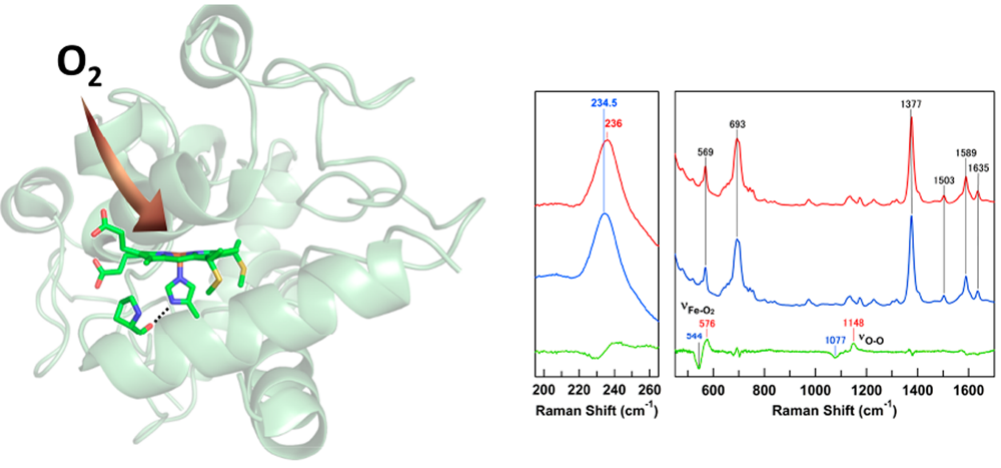

The peroxidase activity of cytochrome (cyt) *c* increases when Met80 dissociates from the heme iron, which is related to the initial cyt *c* membrane permeation step of apoptosis. Met80-dissociated cyt *c* can form an oxygenated species. Herein, resonance Raman spectra of Met80-depleted horse cyt *c* (M80A cyt *c*) were analyzed to elucidate the heme ligand properties of Met80-dissociated cyt *c*. The Fe–His stretching (ν_Fe–His_) mode of ferrous M80A cyt *c* was observed at 236 cm^–1^, and this frequency decreased by 1.5 cm^–1^ for the ^15^N-labeled protein. The higher ν_Fe–His_ frequency of M80A cyt *c* than of other His-ligated heme proteins indicates strong heme coordination and the imidazolate character of His18. Peaks attributed to the Fe–O_2_ stretching (ν_Fe–O_2__) and O–O stretching (ν_O–O_) modes of the oxygenated species of M80A cyt *c* were observed at 576 and 1148 cm^–1^, respectively, under an ^16^O_2_ atmosphere, whereas the frequencies decreased to 544 and 1077 cm^–1^, respectively, under an ^18^O_2_ atmosphere. The ν_Fe–O_2__ mode of *Hydrogenobacter thermophilus* (HT) M59A cyt *c*_552_ was observed at 580 cm^–1^ under an ^16^O_2_ atmosphere, whereas the frequency decreased to 553 cm^–1^ under an ^18^O_2_ atmosphere, indicating that relatively high ν_Fe–O_2__ frequencies are characteristic of *c*-type cyt proteins. By comparison of the simultaneously observed ν_Fe–O_2__ and ν_O–O_ frequencies of oxygenated cyt *c* and other oxygenated His-ligated heme proteins, the frequencies tend to have a positive linear relationship; the ν_Fe–O_2__ frequency increases when the ν_O–O_ frequency increases. The imidazolate character of the heme-coordinated His and strong Fe–O and O–O bonds are characteristic of cyt *c* and apparently related to the peroxidase activity when Met80 dissociates from the heme iron.

## 1 Introduction

Cytochrome (cyt) *c* is a heme protein that shuttles electrons from the cytochrome *bc*_1_ complex to cytochrome *c* oxidase in mitochondria. Cyt *c* also plays a key role in apoptosis by its release into the cytosol upon permeabilization of the mitochondrial outer membrane.^1,2^ Cyt *c* contains three long α-helices, and its heme is bound to the polypeptide chain by covalent bonds with the sulfur atoms of two cysteine residues. Histidine (His18) and methionine (Met80) are coordinated to the heme iron of cyt *c* in its native state,^3,4^ whereas interactions between the N- and C-termini of cyt *c* are thought to be broken upon dissociation of Met80 from the heme iron when it interacts strongly with cardiolipin (CL) in the membrane.^5,6^

The peroxidase activity of cyt *c* increases upon dissociation of Met80 from the heme ion.^6^ The redox potential shifts negatively by 350–400 mV and the peroxidase activity increases dramatically upon the interaction of cyt *c* with CL,^7^ where cyt *c* oxidizes CL.^8,9^ Oxidation of CL enhances the release of cyt *c* from the intermembrane space, inducing apoptosis.^6,10^ In the crystal structure of a trimethyllysine 72-to-alanine mutant of yeast iso-1 cyt *c*, Met80 has been shown to swing out of the heme crevice and be replaced with a water molecule, resulting in a higher peroxidase activity than that of the wild-type protein.^11^ The Met80–heme iron bond is also perturbed in the ferric domain-swapped cyt *c* dimer, which exhibits a higher peroxidase activity than the monomer.^12−14^ In the Tyr67 variants of cyt *c*, the Met80–heme iron bond is disrupted, and the peroxidase activity increases.^15−17^ The peroxidase activity of the P76C variant of cyt *c* increases by ∼13-fold relative to the activity of the wild-type protein.^18^ The Ile81 variants also show a significant enhancement in peroxidase activity, particularly below pH 7, by decreasing the stability to acid unfolding and increasing the accessibility of high-spin heme, which is thought to be essential for peroxidase activity.^19,20^ Acidic conditions cause Lys replacement by a water ligand in Met-SO cyt *c* (p*K*_a_ = 6.3 ± 0.1), resulting in an increase in the intrinsic peroxidase activity.^21^ The peroxidase activity of cyt *c* at the membrane interface of a tetraoleoyl CL (TOCL)-enriched large unilamellar vesicle is higher than that estimated from the interface proton concentration, supporting the hypothesis that upon interaction with TOCL, cyt *c* opens the heme crevice to substrates.^22^ Met80 is selectively oxidized, and a ferryl species (Compound I) forms during the reaction of ferric cyt *c* with a peroxide, *meta*-chloroperbenzoic acid, in the presence of CL-containing liposomes.^23^ In carboxymethylated cyt *c*, Met80 is carboxymethylated, and the Met80-heme iron bond is cleaved; thus, carboxymethylated cyt *c* reacts with excess H_2_O_2_, giving rise to an absorption band at 628 nm, characteristic of Compound I.^24^

Destabilization of the heme–Met80 bond also allows binding of other external ligands, such as carbon monoxide to the ferrous heme iron^25,26^ and nitric oxide and cyanide ion to the ferric heme iron of cyt *c*.^27,28^ Replacement of Met80 with alanine in cyt *c* creates a binding site for molecular oxygen (O_2_) to the heme iron, producing a stable oxygenated complex.^25,29^ When Met80 dissociates from the heme iron of cyt *c* in the presence of a reducing agent, Met80 is oxidized site-specifically by the formation of an oxygenated and subsequent Compound I-like species.^30,31^ The ligand binding property of yeast iso-1 M80A cyt *c* has been investigated by EPR and optical spectroscopic measurements, and the results showed that H_2_O is coordinated to the heme at low pH and is replaced by a hydroxide ion upon increasing the pH (p*K*_a_ ≈ 5.6).^32^ According to surface-enhanced Raman spectroscopy studies combined with molecular dynamics simulation, ferrous cyt *c* undergoes autoxidation with O_2_ and a relatively large conformational alteration after binding to CL, inducing the higher peroxidase activity of cyt *c* and higher permeability of the CL membrane than those induced by ferric cyt *c*.^33^

The resonance Raman spectra of heme proteins provide detailed information on the heme coordination characteristics.^34−37^ For example, on the basis of resonance Raman spectra in the low-frequency region, it has been suggested that the heme of M80A human cyt *c* has a ^–^OH-Fe-His coordination,^38^ as reported for iso-1 M80A cyt *c*.^32^ To obtain more detailed information on the heme coordination character of cyt *c*, we performed resonance Raman studies on the ferrous and oxygenated species of horse cyt *c* in which the heme-coordinating Met was mutated to Ala. We observed high Fe–His stretching (ν_Fe–His_), Fe–O_2_ stretching (ν_Fe–O_2__), and O–O stretching (ν_O–O_) frequencies compared to those of other His-ligated heme proteins, which are presumably related to the peroxidase activity and apoptotic properties of cyt *c*.

## 2 Materials and Methods

### 2.1 Preparation of Horse M80A cyt *c* and HT M59A cyt *c*_552_

Amino acid substitution of Met80 to Ala in horse cyt *c* was performed by PCR-based *in vitro* mutagenesis of the original horse cyt *c*-encoding plasmid vector^39^ using H-M80A-F and H-M80A-R primers (Table S1) and PrimeSTAR Max DNA polymerase (Takara). DNA sequencing was carried out with a BigDye Terminator v3.1 cycle sequencing kit (Applied Biosystems, Inc., Foster City, CA) and an ABI 3100 Avant generic analyzer (Applied Biosystems, Inc.). Recombinant M80A cyt *c* was overexpressed using *Escherichia coli* (*E. coli*) Rosetta 2(DE3) pLysS cells (Novagen) with a method similar to that reported previously.^40^ To obtain ^15^N-labeled M80A cyt *c*, *E*. *coli* cells were grown in M9 minimum medium with ^15^NH_4_Cl (CIL, ^15^N 98 atom %). M80A cyt *c* was purified according to previously reported methods.^40,41^

Amino acid substitution of Met59 to Ala in HT cyt *c*_552_ was also performed by PCR-based *in vitro* mutagenesis of the original HT cyt *c*_552_-encoding plasmid vector using HT-M59A-F and HT-M59A-R primers (Table S1). The plasmid DNAs were enhanced with DH5α cells and transformed into *E. coli* JCB387 containing the pEC86 plasmid DNA.^42^ HT M59A cyt *c*_552_ was purified as reported previously.^43^

### 2.2 Spectroscopic Measurements

Resonance Raman scattering of horse M80A cyt *c* and HT M59A cyt *c*_552_ was performed with excitation at 405.1 nm with a diode laser (ONDAX SureLock, Model LM-403-PLR-40-2) at room temperature, and detection was performed with a liquid nitrogen-cooled CCD (Roper Scientific, 7375-0001) attached to a single polychromator (SPEX, 750M). The slit width of the polychromator was set to 110 μm. A holographic notch filter (Kaiser Optical Systems, Inc.) was used to reduce the Rayleigh scattering. The laser power was adjusted to 3.5 or 3.8 mW at the sample point. A spinning cell was used to avoid photoreduction and sample damage. Ferrous horse M80A cyt *c* and HT M59A cyt *c*_552_ in 50 mM potassium phosphate buffer, pH 7.0, were obtained by the addition of dithionite (final concentration, ∼5 mM) to the ferric protein solution under a nitrogen atmosphere. The ^16^O_2_ complex of M80A cyt *c* or M59A cyt *c*_552_ was obtained by dilution of a ferrous M80A cyt *c* (400 μM) or an HT M59A cyt *c*_552_ (800 μM) solution of 50 mM potassium phosphate buffer, pH 7.0, 40 times with 50 mM potassium phosphate buffer, pH 7.0, saturated with ^16^O_2_. The ^18^O_2_ complex of M80A cyt *c* was obtained by using an ^18^O_2_ (ICON, ^18^O 95 atom %)-saturated buffer instead of an ^16^O_2_-saturated buffer. Raman shifts were calibrated with indene and CCl_4_. The accuracy of the peak positions of the Raman bands was ±1 cm^–1^. Optical absorption spectra of oxygenated M80A cyt *c* and M59A cyt *c*_552_ were obtained with a UV-2450 spectrophotometer (Shimadzu, Japan) using a 1 cm path length quartz cell at 25 °C. The autoxidation rate constant of oxygenated M59A cyt *c*_552_ at 25 °C was obtained by least-squares fitting of the absorbance change at 399 nm with an exponential curve using the Igor Pro 6.0 software (WaveMetrics, Portland). For the dihedral angle (Φ) formed by the imidazole plane and the nearest N(pyrrole)–Fe–N(pyrrole) plane, the values for peroxidases and cyt *c*′ were taken from ref (44). The Φ angle of horse cyt *c* was calculated from the crystal structure of wild-type horse cyt *c* (PDB ID code: 1HRC).

## 3 Results

**Figure 1:**
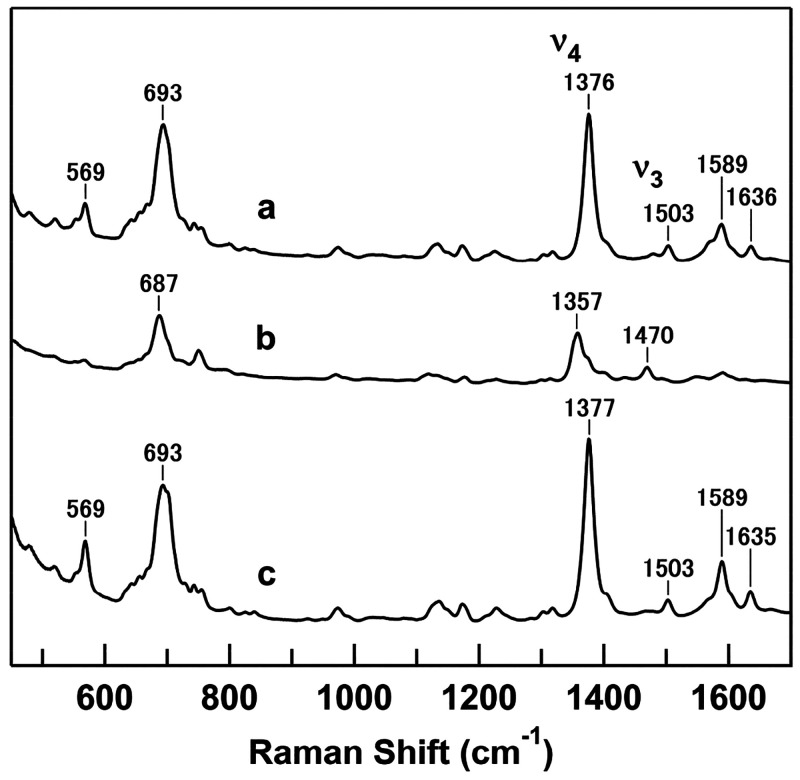
Resonance Raman spectra of ferric (curve a), ferrous (curve b), and oxygenated (curve c) horse M80A cyt *c*. Measurement conditions: cyt *c* concentration, 20 μM; solvent, 50 mM potassium phosphate buffer, pH 7.0; excitation, 405.1 nm; laser power, 3.5 mW; accumulation time, (a,b) 5 min, (c) 20 min; room temperature.

Resonance Raman spectra of horse M80A cyt *c* were obtained to gain insight into the heme coordination character. Some Raman vibration modes of heme proteins are known as marker bands. For example, the high-intensity ν_4_ band at 1355–1375 cm^–1^ is known as an oxidation state marker band. This band is observed at 1370–1375 and 1355–1362 cm^–1^ for ferric and ferrous hemes, respectively.^34−37^ The ν_3_ band is also sensitive to the oxidation and spin states. It is observed at 1480–1483 and 1502–1507 cm^–1^ for high-spin (five-coordinate) and low-spin ferric species, respectively, and at 1472–1473 and 1490–1493 cm^–1^ for high-spin and low-spin ferrous species, respectively. For ferric and ferrous M80A cyt *c*, ν_4_ bands were observed at 1376 and 1357 cm^–1^, respectively (Figure 1, curves a and b). These frequencies were consistent with the traditional frequencies of the corresponding oxidation states. The ν_3_ band was observed at 1503 cm^–1^ for ferric M80A cyt *c*, characteristic of a low-spin ferric state. The ν_4_ and ν_3_ bands are observed at 1373 and 1504 cm^–1^, respectively, for hydroxide ion-bound metmyoglobin under alkaline conditions.^45^ Thus, a hydroxide ion is presumably coordinated to the heme iron in ferric M80A cyt *c*. For ferrous M80A cyt *c*, the ν_3_ band was observed at 1470 cm^–1^, characteristic of a high-spin heme. The ν_4_ and ν_3_ bands have been observed at 1355 and 1472 cm^–1^, respectively, for ferrous deoxy myoglobin (Mb).^34^ Considering these results, ferrous M80A cyt *c* may possess oxidation and spin states similar to those of ferrous Mb due to the removal of heme-coordinating Met, allowing O_2_ to bind to the heme iron. For oxygenated M80A cyt *c*, the ν_4_ band was observed at 1377 cm^–1^ (Figure 1, curve c). The ν_4_ band has been observed at 1377–1378 cm^–1^ for oxygenated hemoglobin (Hb),^46^ Mb, and horseradish peroxidase,^47^ where the increase in the ν_4_ frequency of the oxygenated species compared to that of the ferrous state has been attributed to a decrease in an electron density of the heme iron by the coordination of O_2_. However, although the oxygenated M80A cyt *c* is relatively stable (Figure S1),^25^ M80A cyt *c* could be slightly reoxidized and affect the spectra during the measurements, as the reoxidized ferric species resonates better than the oxygenated species upon 405 nm excitation.

**Figure 2:**
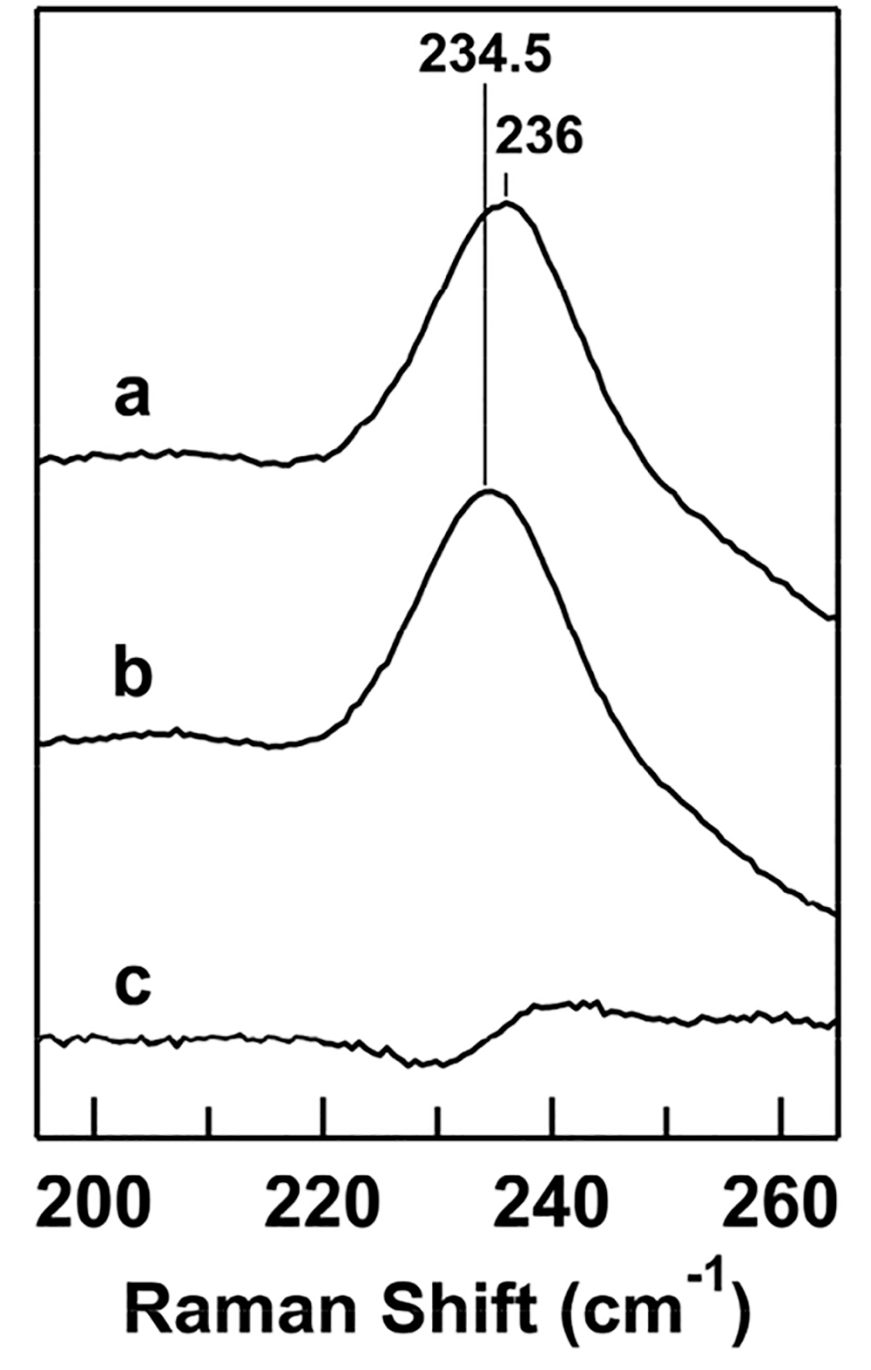
Resonance Raman spectra of natural abundant (curve a) and ^15^N-labeled (curve b) ferrous horse M80A cyt *c* and their difference spectrum (natural abundant minus ^15^N-labeled) (curve c). Measurement conditions are the same as those in Figure 1 except for the accumulation time (10 min).

The resonance Raman spectra of ferrous horse M80A cyt *c* were obtained to investigate the Fe-His bond property of cyt *c*. The band at 236 cm^–1^ in the spectrum of natural-abundance ferrous M80A cyt *c* shifted to a lower frequency of approximately 1.5 cm^–1^ in the spectrum of the ^15^N-labeled protein, allowing assignment of this band to the ν_Fe–His_ mode (Figure 2).

**Figure 3:**
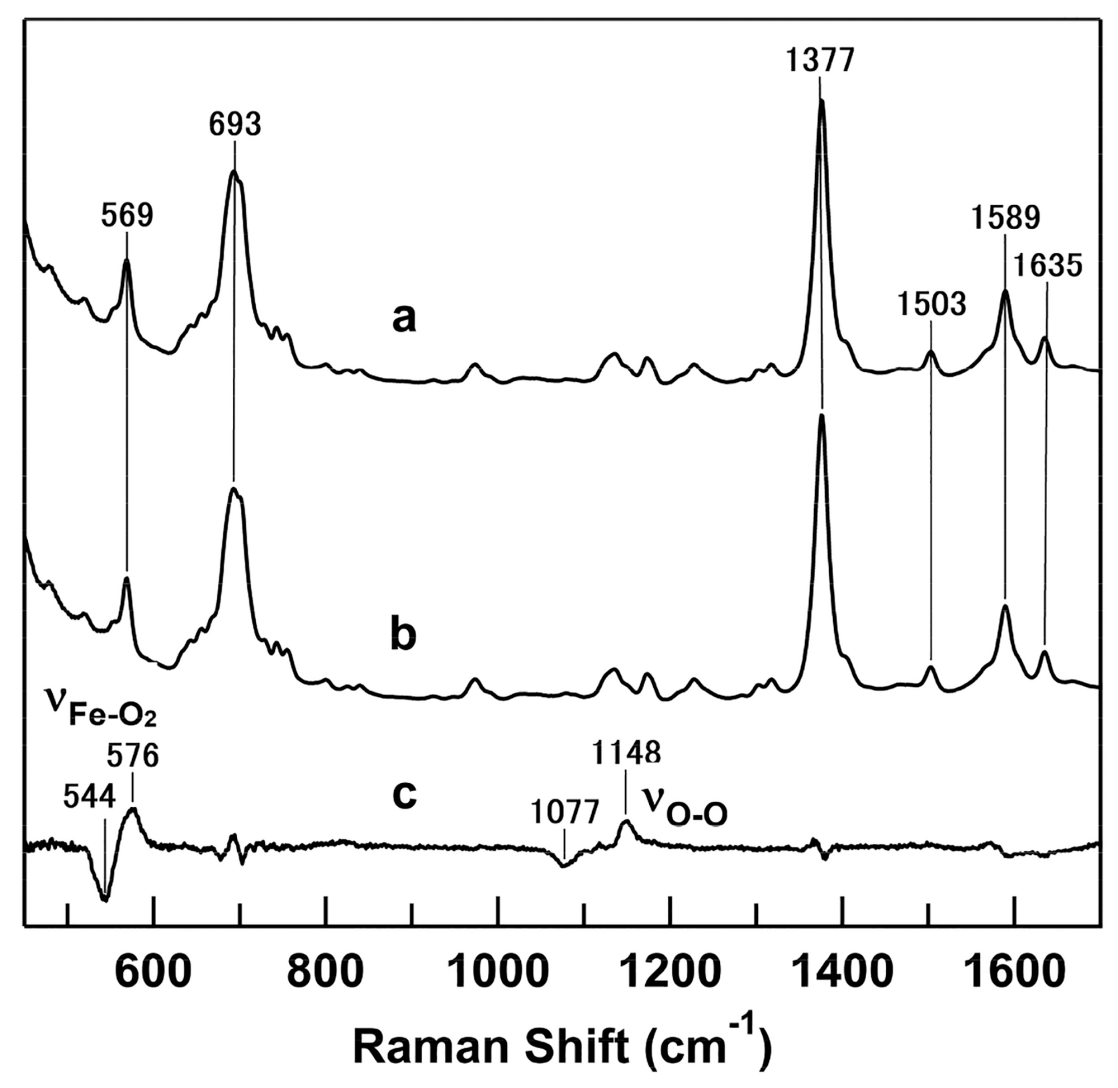
Resonance Raman spectra of oxygenated horse M80A cyt *c* under ^16^O_2_ (curve a) and ^18^O_2_ (curve b) atmosphere and their difference spectrum (^16^O_2_-adduct minus ^18^O_2_-adduct) (curve c). The intensities of the difference spectra are expanded ten times. Measurement conditions are the same as those in Figure 1. The accumulation time is 20 min.

The resonance Raman spectra of oxygenated horse M80A cyt *c* were obtained to gain information about the Fe–O_2_ bond properties when O_2_ was bound to cyt *c*. Although no apparent difference was observed between the spectra of oxygenated M80A cyt *c* under ^16^O_2_ and ^18^O_2_ atmospheres, two O_2_ isotope-dependent patterns were observed in the difference spectrum between the spectra obtained under ^16^O_2_ and ^18^O_2_ atmospheres (Figure 3). The band at 576 cm^–1^ in the spectrum obtained under an ^16^O_2_ atmosphere frequency shifted to 544 cm^–1^ in the spectrum obtained under an ^18^O_2_ atmosphere. Another band at 1148 cm^–1^ obtained in the spectrum under an ^16^O_2_ atmosphere frequency shifted to 1077 cm^–1^ in the spectrum obtained under an ^18^O_2_ atmosphere. From the frequencies and isotope-shift characteristics, the 576 and 1148 cm^–1^ bands were assigned to the ν_Fe–O_2__ and ν_O–O_ modes, respectively. However, the isotopic frequency shift of the ν_Fe–O_2__ band (32 cm^–1^) was larger than that expected for a simple harmonic diatomic oscillator (25 cm^–1^). A similar large isotopic ν_Fe–O_2__ frequency has been observed for oxygenated truncated Hb, suggesting a significantly bent Fe–O–O moiety.^48^ Additionally, it has been recently suggested on the basis of DFT calculations that the O_2_-sensitive band at approximately 570 cm^–1^ corresponds to a significant Fe–O–O bending property,^49^ which may cause a large isotopic shift for ^18^O.

**Figure 4:**
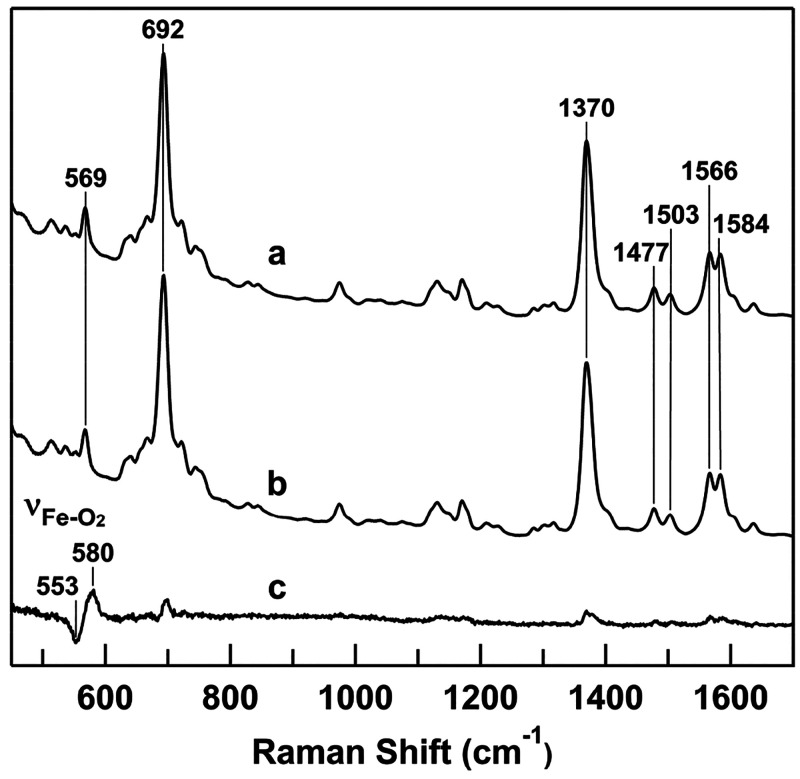
Resonance Raman spectra of oxygenated *Hydrogenobactor thermophilus* M59A cyt *c*_552_ under ^16^O_2_ (curve a) and ^18^O_2_ (curve b) atmospheres and their difference spectrum (^16^O_2_-adduct minus ^18^O_2_-adduct) (curve c). The intensities of the difference spectra are expanded ten times. Measurement conditions: cyt *c*_552_ concentration, 10 μM; solvent, 50 mM potassium phosphate buffer, pH 7.0; excitation, 405.1 nm; laser power, 3.8 mW; accumulation time, 10 min; room temperature.

The resonance Raman spectra of oxygenated HT M59A cyt *c*_552_ were obtained to investigate whether the high ν_Fe–O_2__ frequency is characteristic of *c*-type cyt proteins. In the difference spectrum of oxygenated HT M59A cyt *c*_552_ under ^16^O_2_ and ^18^O_2_ atmospheres, a difference pattern was observed, indicating a frequency shift for the band at 580 cm^–1^ in the spectrum obtained under an ^16^O_2_ atmosphere to 553 cm^–1^ in the spectrum obtained under an ^18^O_2_ atmosphere (Figure 4). However, we could not observe the ν_O–O_ Raman band for oxygenated HT M59A cyt *c*_552_. The autoxidation of M59A cyt *c*_552_ occurred with a rate constant of 0.025 ± 0.001 min^–1^ at 25 °C, whereas the oxygenated M80A cyt *c* was relatively stable as previously reported (Figure S1).^25^ Thus, slight autoxidation may have occurred during the oxygenated HT M59A cyt *c*_552_ Raman measurements, resulting in a slightly lower intensity for the ν_Fe–O_2__ band in the oxygenated HT M59A cyt *c*_552_ spectra compared to that in the M80A cyt *c* spectra (Figures 3 and 4).

## 4 Discussion

**Figure 5:**
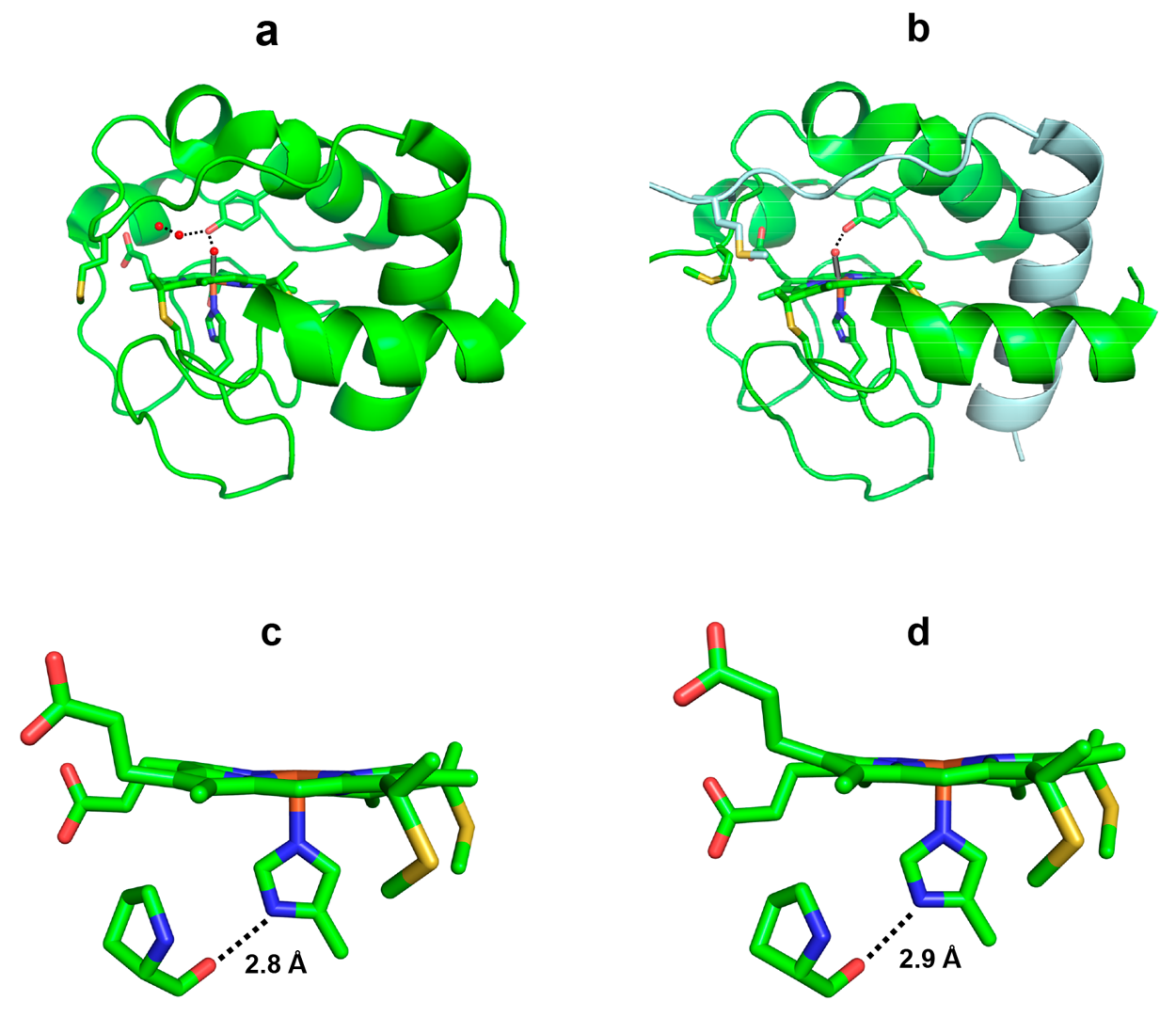
Structures of (a,c) yeast iso-1 K72A cyt *c* (PDB ID code: 4MU8) and (b,d) horse cyt *c* dimer (PDB ID code: 3NBS): (a,b) hydrogen bonding network around the heme and Tyr67, (c,d) hydrogen bond of heme-coordinating His. Met80 is dissociated from the heme iron in yeast iso-1 K72A cyt *c* and horse cyt *c* dimer. The heme and side chains of Cys14, Cys17, His18, Tyr67, and Met80 are shown as stick models. The water molecule or hydroxide ion coordinated to the heme iron and water molecules in the hydrogen bonding network are shown as red spheres. Each protomer of the horse cyt *c* dimer is depicted in green or light blue. The nitrogen atoms of the heme-coordinating His18 and the hemes are shown in blue, the oxygen atoms of Tyr67 and the hemes are shown in red, and the sulfur atoms of Cys14, Cys17, and Met80 are shown in yellow.

The ν_Fe–His_ frequency was detected at 236 cm^–1^ for M80A cyt *c* (Figure 2). The ν_Fe–His_ frequency has been detected at 220 cm^–1^ for myoglobin (Mb)^50^ and at 215 and 221 cm^–1^ for T- and R-states of hemoglobin (Hb),^51^ respectively. The ν_Fe–His_ frequencies of peroxidases, which have an imidazolate ligand, are detected at higher frequencies in the range 240–250 cm^–1^.^52,53^ The relatively high ν_Fe–His_ frequency for peroxidases has been attributed to the strong hydrogen bonding of the coordinated His to the surrounding amino acid residues.^52,53^ Hydrogen bonding between the coordinated His and the backbone carbonyl oxygen atom of Pro30 has been observed in yeast iso-K72A cyt *c* (PDB ID code: 4MU8) and domain-swapped dimeric horse cyt *c* (PDB ID code: 3NBS), in both of which Met80 is dissociated from the heme iron (Figure 5c,d), consistent with a high ν_Fe–His_ frequency for M80A cyt *c*. The relationship of the ν_Fe–His_ frequency in 5-coordinate high-spin ferrous heme complexes to the dihedral angle Φ formed by the imidazole plane and the nearest N(pyrrole)–Fe–N(pyrrole) plane exhibits a linear correlation, from which the property of the axial ligand can be estimated.^44^ The plots of ν_Fe–His_ frequency vs Φ for horse M80A cyt *c* and HT M59A cyt *c*_552_ were consistent with the plots for proteins having His coordination with an imidazolate character, supporting the hypothesis that the heme-coordinated His residues of horse cyt *c* and HT cyt *c*_552_ form relatively strong hydrogen bonding to the surrounding amino acid residues (Figure S2).

The ν_Fe–O_2__ Raman band of a heme protein was first detected for oxygenated Hb at 567 cm^–1^.^54^ The ν_Fe–O_2__ frequencies have also been observed at similar frequencies for oxygenated Mb (571 cm^–1^),^55^ heme oxygenase (565 cm^–1^),^56^ and the O_2_-bound intermediates of cyt *c* oxidase (571 cm^–1^)^57−59^ and indoleamine 2,3-dioxygenase (IDO) (564 cm^–1^).^60^ In these proteins, His coordinates to the heme iron as an axial ligand. The ν_Fe–O_2__ band of cyt P450 has been observed at 541 cm^–1^, which is lower than the ν_Fe–O_2__ frequencies of oxygenated His-coordinated heme proteins.^61^ However, the ν_Fe–O_2__ frequencies of M80A cyt *c* and M59A cyt *c*_552_ were both higher than the traditional ν_Fe–O_2__ frequencies of oxygenated heme proteins with His-ligation (Figures 3 and 4). As seen from a comparison of five-coordinate and six-coordinate oxygenated model heme compounds, the ν_Fe–O_2__ frequencies of six-coordinate oxygenated heme compounds are higher than those of five-coordinate oxygenated heme compounds.^62^ On the other hand, the ν_Fe–O_2__ frequency has been shown to decrease by decreasing the electron density of the heme iron by introducing strongly electron-withdrawing CF_3_ groups to the heme.^63^ Additionally, the ν_Fe–His_ frequency of cyt *c* was higher than those of other His-coordinated heme proteins, such as Mb and Hb (Figure 2). These results indicate that the axial His ligand is coordinated to the heme iron more strongly in *c*-type cyt proteins than in other His-coordinated heme proteins, thus increasing the electron density of the heme iron. However, other factors may affect the ν_Fe–His_ and ν_Fe–O_2__ frequencies, since the ν_Fe–His_ frequency of IDO is relatively high, whereas the ν_Fe–O_2__ frequency is similar to those of Mb and Hb.^60,64^

The ν_O–O_ Raman band was observed for oxygenated M80A cyt *c*, which is rather specific for an oxygenated heme protein with His ligation (Figure 3). The enhancement of the ν_O–O_ mode has been suggested to be related to a dynamic hydrogen bond network between the heme-bound O_2_ and its surrounding environment.^60^ Tyr67 forms a hydrogen bond with the bound oxygen of a H_2_O/OH^–^ ion (3.2 Å) in yeast iso-1 K72A cyt *c* (PDB ID code: 4MU8), in which Met80 is dissociated from the heme ion, and additional water molecules exist around the heme open space (Figure 5a,b).^11^ Tyr67 also forms a hydrogen bond to the oxygen atom of a hydroxide ion bound to the heme (2.9 Å) in the domain-swapped cyt *c* dimer (PDB ID code: 3NBS), where Met80 is dissociated from the heme iron.^12^ The enhancement of the ν_O–O_ Raman band for oxygenated horse M80A cyt *c* indicates a dynamic hydrogen bond network between the heme-bound O_2_ and its surrounding environment. For HT cyt *c*_552_, there is no amino acid similar to Tyr67 in cyt *c* that can interact with the heme-bound O_2_, and the ν_O–O_ Raman mode is not observed (Figure 4).

**Figure 6:**
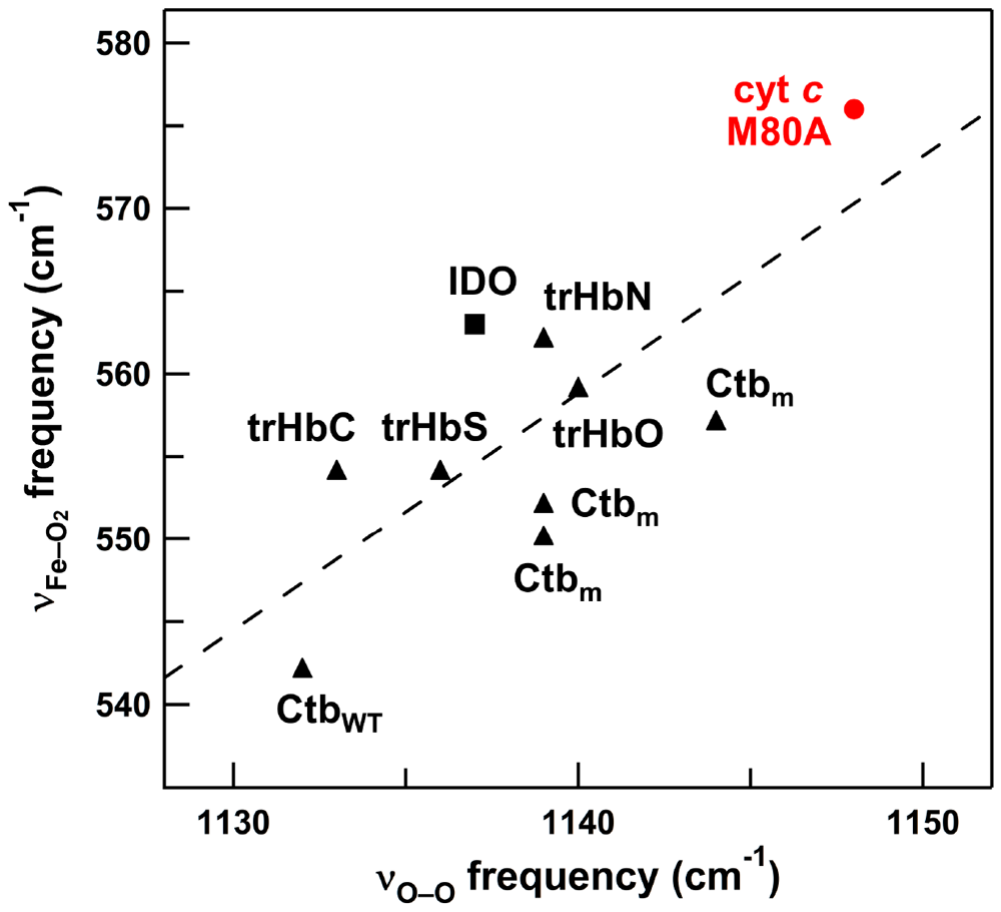
Plots of ν_Fe–O_2__ versus ν_O–O_ frequencies of oxygenated heme proteins with His ligation simultaneously observed by resonance Raman spectroscopy. The plot for M80A cyt *c* is shown as a red circle. The data of truncated Hbs^48,71−73^ and IDO^60^ are shown as triangles and a square, respectively: **trHbN**, *Mycobacterium tuberculosis* Hb; **trHbO**, *Mycobacterium tuberculosis* Hb; **Ctb**_**WT**_, wild-type *Campylobacter jejuni* Hb; **Ctb**_**m**_, mutant *Campylobacter jejuni* Hb; **trHbC**, *Chlamydomonas eugametos* Hb; **trHbS**, *Synechocystis* PCC6803 Hb; **IDO**, indoleamine 2,3-dioxygenase. The least-squares fitted line of the plots is also shown as a dashed line.

For CO-bound heme proteins, a negative linear correlation between the Fe–CO (ν_Fe–CO_) and C–O (ν_C–O_) stretching frequencies has been well characterized.^62,65−69^ The ν_Fe–CO_ frequency increases as the ν_C–O_ frequency decreases, which has been attributed to the Fe–C–O bonds being dominated by the back-bonding of Fe d_π_ electrons into the CO π* orbitals. It has also been reported that the ν_Fe–O_2__ and ν_O–O_ frequencies of oxygenated heme complexes (mainly 5-coordinate) exhibit a negative linear correlation, which is more sensitive than that between ν_Fe–CO_ and ν_C–O_ frequencies, owing to the higher sensitivity of the Fe–O_2_ bond to electronic influences that affect metal-to-ligand back-bonding than that of the Fe–CO bond.^62,70^ On the other hand, a positive linear correlation between the ν_Fe–O_2__ and ν_O–O_ frequencies has been reported for Ctb and its mutants; i.e., the ν_Fe–O_2__ frequency increases when the ν_O–O_ frequency increases.^48^ Perturbation of the σ-bonding system has been suggested to explain this positive correlation.^48^ Although observation of the ν_Fe–O_2__ and ν_O–O_ Raman frequencies for the same heme protein is limited, the simultaneously observed ν_Fe–O_2__ and ν_O–O_ Raman frequencies of horse M80A cyt *c* were plotted together with the simultaneously observed ν_Fe–O_2__ and ν_O–O_ Raman frequencies of oxygenated heme proteins, including Ctb and IDO (Figure 6).^48,60,71−73^ Even though the species differ, the ν_Fe–O_2__ and ν_O–O_ Raman frequencies showed a positive linear correlation. However, the ν_Fe–O_2__ frequency was more sensitive to the ν_O–O_ frequency than was the ν_Fe–CO_ frequency to the ν_C–O_ frequency; the ν_Fe–O_2__/ν_O–O_ slope (+1.43) was steeper than the ν_Fe–CO_/ν_C–O_ slope of 6-coordinated CO-bound model compounds (−0.73).^62^

The oxygenated species of horse M80A cyt *c* was more stable than that of HT M59A cyt *c*_552_ (Figure S1). HT is a bacterium that does not undergo apoptosis; thus, the high stability of the oxygenated species of horse M80A cyt *c* may be advantageous to peroxidase activity and apoptotic properties. The residue at amino acid position 67 has been reported to affect the peroxidase activity of cyt *c*. For example, the peroxidase activity in the Y67H/M80A double mutant increased slightly with respect to that of the wild-type protein.^38^ Significant peroxidase activity was observed for the Y67R mutant, highlighting the role of Arg as a base, which may act as an acid to cleave the O–O bond in H_2_O_2_.^17^ The high stability of the oxygenated species of M80A cyt *c* may be attributed to Tyr67,^25^ and the faster autoxidation rate for oxygenated M59A cyt *c*_552_ than for M80A cyt *c* is apparently due to the lack of an amino acid residue that can stabilize the bound O_2_. Tyr67 may stabilize the oxygenated species of cyt *c* and play an important role in increasing the peroxidase activity of cyt *c*. Although oxygenated M59A cyt *c*_552_ exhibited faster autoxidation than oxygenated M80A cyt *c*, oxygenated M59A cyt *c*_552_ was more stable than other oxygenated heme proteins without a distal His, which may be due to the strong Fe–O bond. The proximal His imidazolate character, relatively strong Fe–His bond, and relatively stable oxygenated species of cyt *c* may be related to its peroxidase character for apoptosis.

## 5 Conclusions

We observed the ν_Fe–His_ Raman band at 236 cm^–1^ for the ferrous form and ν_Fe–O_2__ and ν_O–O_ Raman bands at 576 and 1148 cm^–1^, respectively, for the oxygenated species of horse cyt *c* by mutating heme-coordinating Met80 to Ala. The ν_Fe–O_2__ Raman band of another methionine-depleted *c*-type cyt, HT cyt *c*_552_, was also detected at 580 cm^–1^. The ν_Fe–O_2__ and ν_O–O_ frequencies of M80A cyt *c* and M59A cyt *c*_552_ were also higher than the corresponding frequencies of other heme proteins with His ligation. The strong His-ligation character and high ν_Fe–O_2__ and ν_O–O_ frequencies were specific for *c*-type cyt proteins, where these characteristics are apparently related to the activation of the bound O_2_ for peroxidase activity and apoptotic properties. Tyr67 plays an important role in stabilizing the oxygenated species of cyt *c*, which may be related to the relatively high peroxidase activity of cyt *c*.

## Supporting Information Available

Nucleotide sequences of the primers; changes in the absorption spectra of oxygenated horse M80A cyt *c* and HT M59A cyt *c*_552_; Φ dependence of the ν(Fe–N(imidazole)) band of 5-coordinate high-spin ferrous heme proteins and complexes (PDF)
