# Supplementary material for: Resonance Raman Studies on Heme Ligand Stretching Modes in Methionine80-Depleted Cytochrome c: Fe–His, Fe–O2, and O–O Stretching Modes
Source: J Phys Chem B. 2023 Mar 15;127(11):2441–9. doi: 10.1021/acs.jpcb.3c00514 (PMC10041640; doi:10.1021/acs.jpcb.3c00514)
Supplement: Supplementary file 1 — jp3c00514_si_001.pdf [file jp3c00514_si_001.pdf]

Supporting Information

Resonance Raman Studies on Heme Ligand Stretching Modes in Methionine80-Depleted Cytochrome *c*: Fe−His, Fe−O_2, and O−O Stretching Modes

Mohan Zhang[a#], Hulin Tai[a§], Sachiko Yanagisawa[b], Masaru Yamanaka[a], Takashi Ogura[b], and Shun Hirota[a*]

[a] *Division of Materials Science, Graduate School of Science and Technology, Nara Institute of Science and Technology (NAIST), 8916-5, Takayama, Ikoma, Nara 630-0192, Japan*

[b] *Graduate School of Life Science, University of Hyogo, Kamigori-cho, Ako-gun, Hyogo 678-1297, Japan*

*E-mail: hirota@ms.naist.jp

Present Addresses:

[#] *School of Chemistry and Chemical and Engineering, Guangxi Minzu University, Nanning 530008, China*

[§] *Department of Chemistry, Yanbian University, Yanji, Jilin 133002, China*

Table of Contents

**Table S1.**	Nucleotide sequences of the primers.	p. S2
**Figure S1.**	Changes in the absorption spectra of oxygenated horse M80A cyt *c* and HT M59A cyt c_{552} by incubation under air.	p. S3
**Figure S2.**	Φ dependence of the ν(Fe-N(imidazole)) band of 5-coordinate high-spin ferrous heme proteins and complexes.	p. S4

Table S1. Nucleotide sequences of the primers.

Primer	Sequence[a]
H-M80A-F	ACAAAAGCGATCTTTGCTGGCATTAAG
H-M80A-R	AAAGATCGCTTTTGTTCCAGGGATGTA
HT-M59A-F	GTTCCCGCGCCTCCTCAAAATGTAACCG
HT-M59A-R	AGGAGGCGCGGGAACAGAACCCCACAC

[a] Underlines indicate the replaced nucleotides.

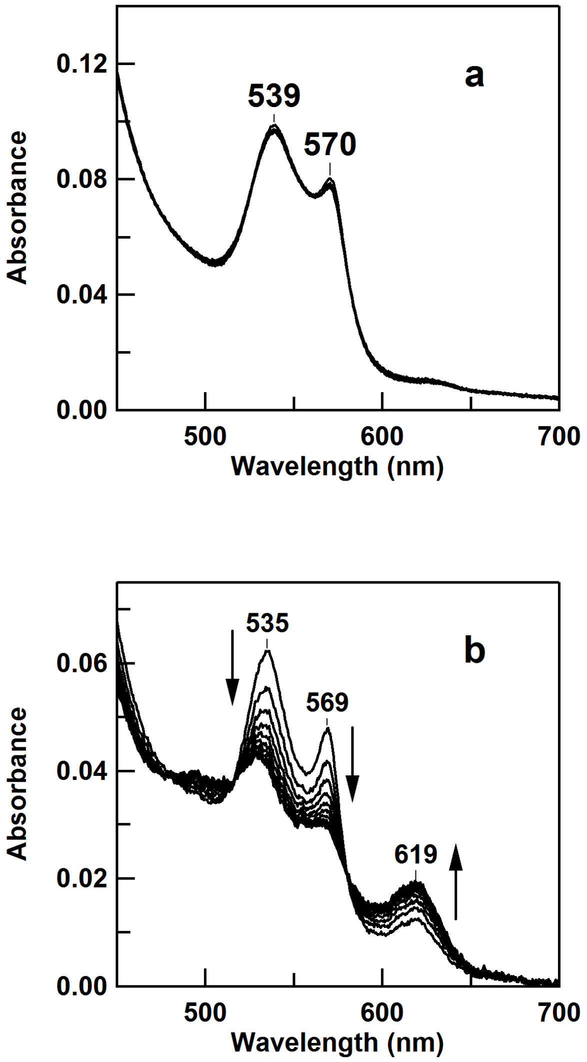

Figure S1. Changes in the absorption spectra of (a) oxygenated horse M80A cyt *c* and (b) HT M59A cyt c_{552} by incubation under air. Experimental conditions: sample concentration, horse M80A cyt *c*, 10 μM, HT M59A cyt c_{552}, 5 μM; solvent, 50 mM potassium phosphate buffer, pH 7.0; temperature, 25 °C; spectra shown, horse M80A cyt *c*, 30 min interval (up to 90 min incubation), HT M59A cyt c_{552}, 10 min interval (up to 90 min incubation).

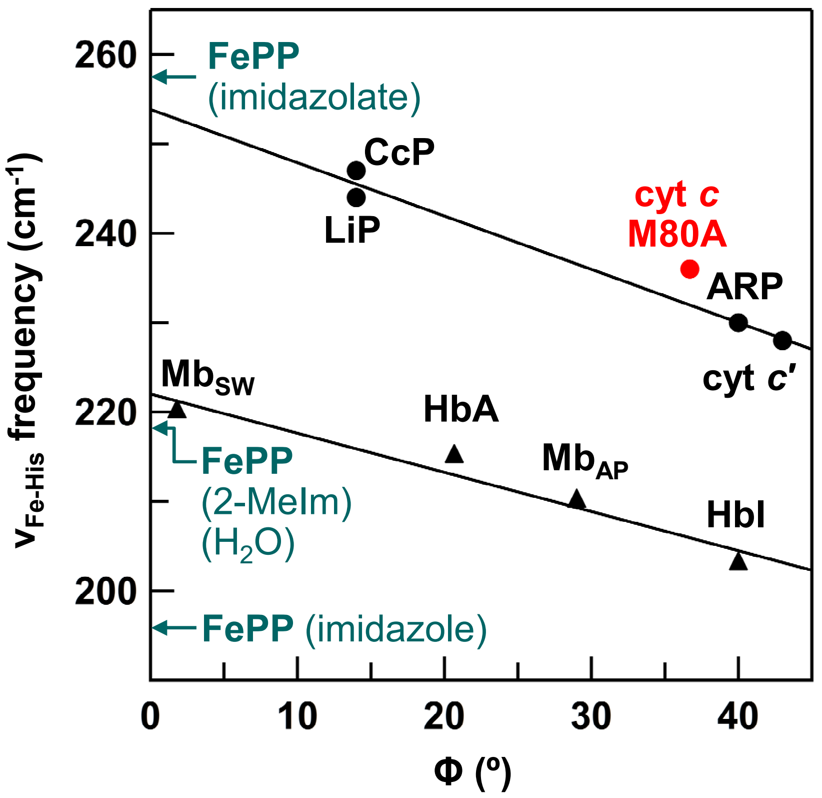

Figure S2. Φ dependence of the ν(Fe-N(imidazole)) band of 5-coordinate high-spin ferrous heme proteins and complexes:[43] **LiP**, lignin peroxidase; **ARP**, *Arthromyces ramosus* peroxidase; **Mb**$_{SW}$, sperm whale myoglobin; **Mb**$_{AL}$, *Aplysia limacine* myoglobin; **HbA**, human hemoglobin A, **HbI**, *Scapharca inaequivalvis* hemoglobin; **cyt *c*′**, *Rhodospirillum rubrum* cytochrome *c*′; **FePP**, Fe(II)-protoporphyrin; 2-MeIm, 2-methylimidazole.
